# Apolipoprotein D Upregulation in Alzheimer’s Disease but Not Frontotemporal Dementia

**DOI:** 10.1007/s12031-018-1217-9

**Published:** 2018-11-22

**Authors:** Surabhi Bhatia, Woojin Scott Kim, Claire E. Shepherd, Glenda M. Halliday

**Affiliations:** 1 Central Clinical School and Brain and Mind Centre, Faculty of Medicine and Health Sciences, The University of Sydney, 94 Mallet Street, Camperdown, NSW Australia; 2 Neuroscience Research Australia, Sydney, NSW Australia; 3 School of Medical Sciences, University of New South Wales, Sydney, NSW Australia

**Keywords:** Apolipoprotein D, Oxidative stress, Neurodegeneration, Frontotemporal dementia, TDP43

## Abstract

Frontotemporal dementia (FTD) and Alzheimer’s disease (AD) are the two common forms of dementia. FTD syndromes are characterized by lobar atrophy (frontotemporal lobar degeneration or FTLD) and the presence of either cellular TDP43 (FTLD-TDP), tau (FTLD-tau), or FUS aggregates, while extracellular β-amyloid plaques and hyperphosphorylated tau tangles develop in AD. Oxidative stress can induce these pathological modifications in disease models, and is thought to play a role in these syndromes. Apolipoprotein D (apoD) is a glial-expressed lipocalin known to protect against oxidative stress, with increased levels in AD, supporting a protective role. The expression of apoD has not been studied in FTLD. This study assesses apoD expression in FTLD-TDP and FTLD-tau in comparison to AD and controls. It also analyzes the effect of apoD on *TARDBP* (TDP43 gene) and β-amyloid precursor protein (*APP*). The expression of apoD was analyzed by Western blotting in FTLD-TDP, FTLD-tau, AD, and control post-mortem brain tissue. An apoD-overexpressing cell model was used to study the impact of increased apoD on *APP* and *TARDBP* expression. We confirm that apoD expression was increased in AD but surprisingly it was not affected in either of the two main pathological forms of FTLD. Under oxidative stress conditions, apoD had no effect on TDP43 expression but it did decrease *APP* expression. This suggests that apoD does not act as a neuroprotective factor in FTLD in the same way as in AD. This could contribute to the more rapid degeneration observed in FTLD.

**Electronic supplementary material:**

The online version of this article (10.1007/s12031-018-1217-9) contains supplementary material, which is available to authorized users.

## Introduction

Apolipoprotein D (apoD) is a 29 kDa highly conserved lipocalin known for its antioxidant and neuroprotective functions (He et al. 2009; Tsukamoto et al. 2013; Dassati et al. 2014). ApoD is known to be upregulated in astrocytes during aging (Loerch et al. 2008; de Magalhaes et al. 2009) and in various neurological disorders including schizophrenia, bipolar disorder (Thomas et al. 2001; Mahadik et al. 2002), stroke (Rassart et al. 2000), Alzheimer’s disease (AD) (Terrisse et al. 1998; Belloir et al. 2001; Glockner and Ohm 2003; Bhatia et al. 2013), Parkinson’s disease (PD) (Ordonez et al. 2006), and Niemann-Pick’s C disease (Suresh et al. 1998). ApoD knockout mouse models have provided evidence that the loss of apoD is associated with motor and cognitive deficits (Ganfornina et al. 2008; Bajo-Grañeras et al. 2011a). The loss of apoD is known to disrupt proteostasis machinery and induce oxidative and inflammatory damage (Thomas et al. 2003; Sanchez et al. 2015). It has been demonstrated that apoD helps to maintain neuronal homeostasis by combatting lipid peroxidation (Ganfornina et al. 2008).

Frontotemporal lobar degeneration (FTLD) and AD are two major forms of dementia. Neuropathologically, TDP43 and tau protein aggregates are present in the majority of FTLD cases (IR and Manuela 2016) while extracellular β-amyloid plaques produced from amyloid precursor protein (APP) and intracellular hyperphosphorylated tau tangles are the hallmarks of AD (Bloom 2014). Oxidative stress is one of the major factors associated with both of these pathological forms of dementia. Oxidative stress response genes are dysregulated in both TDP43-overexpressing mice (Tsuiji et al. 2017) and human brain post-mortem tissues with FTLD pathology (Schweitzer et al. 2006). Moreover, under induced oxidative stress, cellular TDP43 undergoes phosphorylation and C terminal fragmentation, characteristic of pathological TDP43 in FTLD (Iguchi et al. 2012). Similarly, increased levels of lipid and protein oxidation products are evident in AD (Lyras et al. 1997; Montine et al. 2005) and these are known to modulate β-amyloid production (Tong et al. 2005; Tamagno et al. 2008; Tan et al. 2013a).

ApoD is upregulated in brain astrocytes and CSF of AD patients (Terrisse et al. 1998; Belloir et al. 2001; Bhatia et al. 2013) and is known to colocalize with β-amyloid plaques (Desai et al. 2005). Studies have demonstrated that the apoD homolog in Drosophila (GLaz) protects against β-amyloid-induced cytotoxicity (Muffat et al. 2008). In addition, transgenic AD mouse models with loss of apoD function exhibit twice the amount of hippocampal β-amyloid plaque load along with alterations in β-amyloid-generating proteins (Li et al. 2015). Increasing evidence suggests the antioxidant and anti-inflammatory role of apoD (Ganfornina et al. 2008; Bajo-Grañeras et al. 2011b; Dassati et al. 2014). We therefore hypothesized that apoD plays a neuroprotective role in FTLD; and to test our hypothesis, we analyzed the expression of apoD in FTLD and compared that to AD and controls. As oxidative stress is known to induce pathological modifications in TDP43 and APP, we also assessed the effect of apoD on *APP* and *TARDBP* (TAR DNA–binding protein) gene expression, responsible for pathological aggregates in AD and FTLD respectively.

Our data suggests that unlike AD, apoD is not increased in FTLD. The cell models suggest that under oxidative stress, apoD protects against increased expression of *APP* while it has no effect on *TARDBP*, which is responsible for TDP43 expression. Therefore, this suggests that apoD expression is differentially regulated in FTLD and AD and that it is possible that apoD is unable to protect against oxidative stress in FTLD.

## Material and Methods

### Human Brain Tissue

Frozen post-mortem brain tissue was obtained with approval from Sydney Brain Bank and NSW Brain Tissue Resource Centre as part of the NSW Brain Banks. Brain tissue from the inferior temporal and superior frontal cortex from 18 FTLD cases, 7 AD cases (5 for inferior temporal region), and 11 controls, all without other neuropathological abnormalities, were used in this study. In particular, both the FTLD and control cases were selected for having no or low AD neuropathologic change. Ten FTLD cases had TDP-43 aggregates (FTLD-TDP) with Type A, B, and C pathologies (Tan et al. 2013b), and 8 FTLD cases had tau aggregates (FTLD-tau) with 4R subtype pathologies (Kovacs 2018). Demographic information for each group is provided in Table 1. Ethics approval for the study was from the University of New South Wales Human Research Ethics Committee.

**Table 1:** Demographic and pathological details of control, FTLD-TDP43, FTLD-tau, and AD cohorts. Values are given as mean ± standard deviation for age and post-mortem delay (PMI)

Group	*N*	Age	PMI (h)	Gender (M/F)
Control	11	79.5 ± 12.1	16.3 ± 6.5	5/6
FTLD-TDP43	10	72.9 ± 13	24 ± 8.7	5/5
FTLD-tau	8	73.9 ± 5.8	12.5 ± 9	4/4
AD	7 (superior frontal)	73.7 ± 7.5	2.3 ± 0.7	3/4

### Sample Preparation

Tris-buffered saline (TBS), SDS-soluble and SDS-insoluble proteins were serially extracted from 100 mg of fresh-frozen tissue from each brain region, as previously described (Bhatia et al. 2013; Murphy et al. 2013). Briefly, tissue was homogenized in ten volumes of TBS homogenization buffer (20 mM Tris, 150 mM NaCl, pH 7.4, 5 mM EDTA, 0.02% sodium azide) containing protease inhibitor cocktail (Roche) using Qiagen tissue lyser (3 × 30 sec, 30 Hz cycles), followed by centrifugation at 100,000×*g* for 1 h at 4 °C, with supernatant collected as the TBS-soluble fraction containing cytosolic proteins. The pellet was resuspended in SDS solubilization buffer (TBS homogenization buffer containing 5% SDS) using 3 × 30 sec, 30 Hz cycles with Qiagen Tissue Lyser, and centrifuged at 100,000×*g* for 30 min at 25 °C, with supernatant collected as the SDS-soluble fraction containing membrane-associated proteins. The pellet obtained by SDS fraction was homogenized in 5 M guanidine hydrochloride (gHCl) and mixed in a mixer at room temperature for 4 h. The lysate was further centrifuged at 100,000×*g* for 30 min at 25 °C, with supernatant collected as the SDS-insoluble (gHCl soluble) fraction. Protein concentration was measured using a bicinchoninic acid assay (Pierce BCA Protein Assay Kit, Thermo Scientific), according to the manufacturer’s instructions. Samples were stored at − 80 °C until use.

### Immunoblotting

Equal concentrations of TBS fraction of the protein extracts (15 μg) were heated with sample buffer (3.2% SDS, 32% glycerol, 0.16% bromophenol blue, 100 mM Tris–HCl, pH 6.8, 8% 2-mercaptoethanol *w*/*v*) and separated on Bio-Rad Criterion Stain-free 4–20% SDS-PAGE gels. The gels were activated for 1 min using Bio-Rad chemiDoc MP imaging system prior to transfer of proteins to a 0.45-μm PVDF membrane. The membranes were imaged for total protein using Bio-Rad chemiDoc MP imaging system. Subsequently, the membranes were blocked with 5% milk powder in TBST for 1 h at room temperature and incubated overnight in apoD primary antibody (Santacruz, sc-373965, 1:2000) prior to protein detection using horseradish peroxidase-conjugated secondary antibodies (Bio-Rad) with enhanced chemiluminescence (Amersham ECL Plus Western Blot Detection System, GE Healthcare). The protein band in each gel lane was normalized to total protein using Bio-Rad image lab software.

### Cell Culture

U87-MG cell line (astroglioma) obtained from ATCC was used as apoD is highly expressed in astrocytic cells in vivo. To obtain an apoD-overexpressing stable cell line, the cells were transfected with human apoD plasmid (obtained from OriGENE) using Lipofectamine 3000 as per manufacture’s protocol and then maintained in EMEM media with 10% fetal bovine serum (FBS) and 0.5 mg/ml Geneticin for 2 weeks. After the initial selection process, the cells were maintained in media with 0.25 mg/ml Geneticin. The cells were plated in a six well dish at the density of 1 × 10^5^cells/ml and treated with 300 μM of H_2_O_2_ for 24 h to induce oxidative stress. The experiments were repeated in triplicates for three times.

### RNA Extraction and qPCR

RNA was extracted from the cells using Relia Prep RNA cell Miniprep system (promega) as per manufacture’s protocol. Analysis of *APP* and *TARDBP* expression was performed using iScript cDNA synthesis Kit and SSO advanced SYBR green mix on Bio-Rad CFX connect system. Amplification was carried out with 40 cycles of 94 °C for 15 s and 60 °C for 1 min. The details of the primers used for the study is as follows-*APP* (F: 5′CCGCTGCTTAGTTGGTGAGTTTGT-3′ and R: 5′-ACGGTGTGCCAGTGAAGATGAGTT-3′), *TARDBP* (F 5′-CGGCCTAGCGGGAAAAGTAA-3′ and R:5′TGGAAACTGGGCTGTAACCG-3′), *β-actin* (F: 5′-GAATTCTGGCCACGGCTGCTTCCAGCT-3′, and R: 5′-AAGCTTTTTCGTGGATGCCACAGGACT-3′) *5.8S* forward 5′-GGTGGATCACTCGGCTCGT-3′, and R: 5′-GCAAGTGCGTTCGAAGTGTC-3′, *Cyclophilin A* (F: 5′-AGGGTTCCTGCTTTCACAGA-3′and R: 5′-GTCTTGGCAGTGCAGATGAA-3′).

All gene expression was normalized to the housekeeper genes *β-actin*, *Cyclophilin A*, and *5.8S*. A no-template control was included for each PCR amplification. The level of expression for each gene was calculated using the comparative threshold cycle (CT) value method with the formula 2^−ΔΔCt^ (where ΔΔCT = ΔCT sample − ΔCTreference).

#### Data Analysis

All statistical analyses for post-mortem apoD expression in human brain were performed using SPSS statistical software using *univariate analysis covarying for age, sex, and gender*, with a *p* value < 0.05 considered significant. The relative expression of apoD in human brain tissue is expressed as a percentage of control. The analysis for cell models was completed using Welch’s *t* test on Prism Graphpad, *p* value < 0.05 was considered significant.

## Results

### ApoD Expression in FTLD and AD

**Fig. 1:**
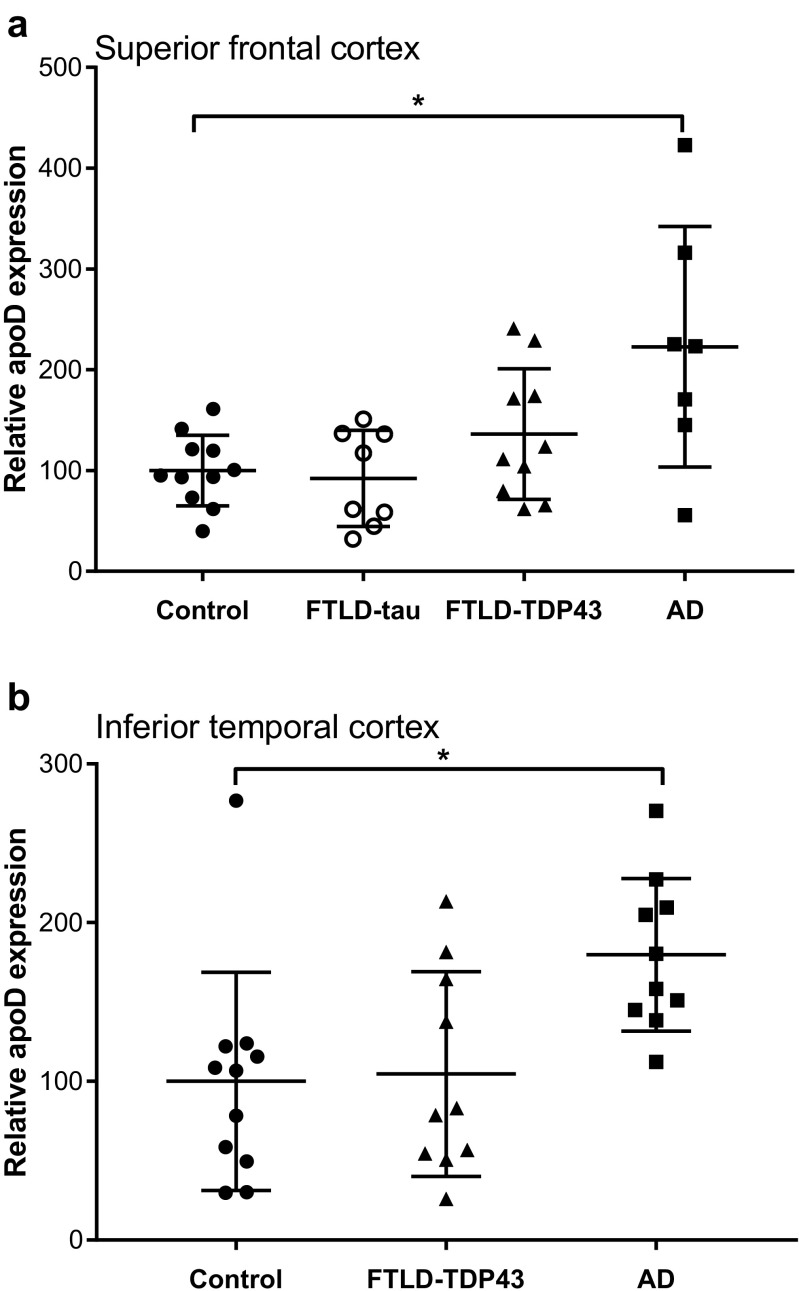
Relative expression of ApoD in TBS-soluble fraction in **a** the superior frontal cortex and **b** inferior temporal cortex. Data represents mean ± SD. Significance is at *p < 0.05

The relative expression of apoD in post-mortem brain tissue of FTLD and AD samples was analyzed in the highly affected superior frontal cortex (SFC) and inferior temporal cortex (ITC) using western blotting. There was no significant difference in soluble apoD levels in FTLD compared to controls in either of the regions analyzed [C vs FTLD-TDP43-SFC; *p* = 0.262, ITC; *p* = 0.490]. This is in contrast to AD where we confirm previous findings of significantly increased apoD protein levels [C vs AD–SFC; *p* = 0.005, ITC; *p* = 0.002] (Belloir et al. 2001; Glockner and Ohm 2003; Desai et al. 2005; Bhatia et al. 2013) (Fig. 1a, b). Comparison of FTLD cases with either TDP43 or tau pathology found no significant differences in the relative soluble apoD levels even in the most affected superior frontal cortex between these groups [FTLD-TDP43 vs FTLD-tau, *p* = 0.367] (Fig. 1a). These data suggest that there are significant differences in the expression of TBS-soluble apoD between FTLD and AD, two different neurodegenerative dementias. As the expression of apoD has not been previously studied in FTLD, we also analyzed the SDS-soluble and SDS-insoluble (gHCl soluble) fraction from the superior frontal cortex in the diseased cohorts. There was no significant difference in apoD expression in either SDS-soluble (Suppl. Fig. 1a,b,c) or gHCL-soluble fractions (Suppl. Fig. 2) in any of the disease groups when compared to controls. Furthermore, we did not detect any specific higher molecular weight aggregates in gHCl fractions in either FTLD or AD compared to controls (Suppl. Fig. 2).

### Effect of apoD on TARDBP and APP Expression Under Oxidative Stress

**Fig. 2:**
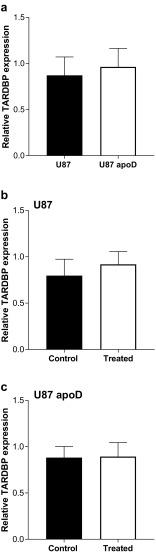
Relative expression of TARDBP in **a** U87- and apoD-overexpressing cell line, **b** in U87 cells, and **c** U87 apoD cells under control vs treated (300 mm H2O2 for 24 h) conditions. Data represents mean ± SD. of *n* = 3 for 3 repeats

**Fig. 3:**
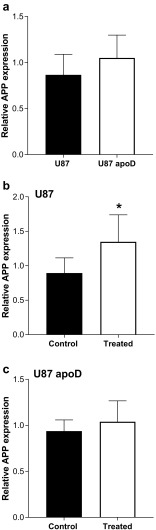
Relative expression of APP in (**a**) U87- and apoD-overexpressing cell line, **b** in U87 cells, and **c** U87 apoD cells under control vs treated (300 mm H2O2 for 24 h) conditions. Data represents mean ± SD of *n* = 3 for 3 repeats. Significance is at *p < 0.05

As mentioned previously, *TARDBP* is the gene encoding the protein that deposits in the pathological aggregates in FTLD-TDP. Therefore, we analyzed the effect of apoD on *TARDBP*. We found that apoD overexpression has no impact on endogenous *TARDBP* expression (Fig. 2a). To determine if increased apoD could impact AD pathology, we analyzed the expression of amyloid precursor protein (*APP*) in apoD-overexpressing U87 cells. We observed no change in expression of *APP* in apoD-overexpressing U87 cells (Fig. 3a) suggesting that the expression of apoD is unlikely to impact on astrocytic β-amyloid production.

Oxidative stress induces pathological modifications in TDP43 (Cohen et al. 2012; Iguchi et al. 2012) and results in increased β-amyloid production (Paola et al. 2000). Therefore, we analyzed the effect of apoD on *TARDBP* and APP expression under oxidative stress. Different concentration and exposure times of hydrogen peroxide (H_2_O_2_) were used to induce oxidative stress to the cells, and based on cell viability assays we used the 300 μM H_2_O_2_, 24-h treatment to induce oxidative stress (suppl. Fig. 3). We found that apoD has no effect on *TARDBP* expression under oxidative stress (Fig. 2b, c). However, we found that in the presence of H_2_O_2_, *APP* expression was significantly upregulated in U87 cells but this effect was not evident in apoD-overexpressing U87 cells (Fig. 3b, c).

## Discussion

This study analyzes the expression of apoD in FTLD, AD, and control brain tissue. As apoD expression in FTLD has not been studied previously, we analyzed the apoD expression in TBS soluble (cytosolic), SDS soluble, and SDS insoluble (gHCl soluble) in FTLD. We found that unlike AD, there was no change in cytosolic apoD expression in either FTLD-TDP or FTLD-tau compared to age-matched controls. Furthermore, there was no difference in the expression of cytosolic apoD between FTLD-TDP and FTLD-tau, two major pathological forms of FTLD (Fig. 1a, b). We also found no difference in apoD expression in SDS-soluble and gHCl-soluble fraction in either FTLD-TDP or FTLD-tau. Furthermore, in contrast to our previous study which has reported apoD dimers in gHCL-soluble fraction (with pathological aggregates) in the hippocampal region in AD (Bhatia et al. 2013), we found no specific apoD aggregates in either FTLD groups or AD in the superior frontal cortex. This suggests that apoD may be present as a dimer only in the highly affected hippocampal region in AD and not in the superior frontal cortex. These results indicate that in contrast to AD where cytosolic apoD expression is increased along with presence of apoD dimers in the highly affected hippocampal region, apoD expression is unaltered and apoD does not seem to aggregate in the most affected superior frontal region in FTLD.

A major difference between AD and FTLD is the deposition of extracellular β-amyloid pathology found in AD, while both have abnormal intracellular protein deposits in the remaining cortical neurons (Ferrer et al. 2014; Irwin et al. 2015; Tan et al. 2015). Therefore, β-amyloid-induced oxidative stress or the presence of β-amyloid itself may be responsible for increased apoD in AD as suggested previously (Martinez et al. 2012).

Apart from extracellular β-amyloid aggregates, AD is also characterized by the presence of intracellular tau deposition. Intracellular tau pathology also underlies a proportion of cases with FTLD (Arai et al. 2006; Leyton and Hodges 2010; Goedert et al. 2012). Our data demonstrate that tau deposition in FTLD does not increase apoD protein expression, suggesting that neuronal tau accumulation of itself does not cause astrocytic upregulation of apoD. Also, the type of tau accumulations in AD versus FTLD differs, with both 3 repeat and 4 repeat tau isoforms incorporated into the neuritic pathology in AD, while only 4 repeat tau isoforms are incorporated into the neuritic pathology of the FTLD-tau cases analyzed in this study (Goedert et al. 1989; Yoshida 2006; Dickson et al. 2011). Astrocytes have 4 repeat tau isoforms (Nishimura et al. 1997; Arai et al. 2001) and it may be that increasing 3 repeat tau protein is a trigger in AD compared with the FTLD.

Alternatively, it may be different in the potential reactivity of astrocytes in these two neurodegenerative dementias that affect apoD expression. Astrocytic apoptosis correlates with the degree of atrophy in FTLD (Broe et al. 2004) and the early loss of astrocytes in FTLD may explain the lack of any potential increase in apoD protein levels in these cases. In fact, reduced astrocytic apoD results in stress-induced astrocytic apoptotic cell death (Bajo-Grañeras et al. 2011a) which may further contribute to the degeneration of these cells in FTLD.

Furthermore, our data suggests that oxidative stress increases β-amyloid production in astrocytes, and that such increased β-amyloid production can be ameliorated by increasing the expression of apoD, in agreement with previous studies (Desai et al. 2005; Martinez et al. 2012; Li et al. 2015). Overall, this data is consistent with an increase expression of astrocytic apoD protein in association with increased oxidation and β-amyloid production in AD. We also found that in the presence of H_2_O_2_, there was no difference in TDP43 expression in U87 cells (Fig. 2), a finding in line with the concept that oxidative stress impacts on TDP43 in a neuron-specific way (Cacabelos et al. 2016), and also in line with our data showing no change in apoD in FTLD-TDP.

Previous studies using apoD knock out mice have shown that apoD specifically protects against oxidation of lipids in the brain while it has no effect on protein oxidation (Ganfornina et al. 2008). A large number of studies have shown increased lipid peroxidation in the AD brain (Pratico et al. 1998; Williams et al. 2006; Fukuda et al. 2009). In comparison, only one study has assessed lipid peroxidation in FTLD and reports that F2 isoprostane (lipid peroxidation marker) levels are not increased in FTLD compared to controls (Yao et al. 2003). The absence of excessive oxidation of brain lipids in FTLD suggests that clearance of lipid peroxidation products may be well preserved in FTLD and therefore, apoD levels remain unaltered. Overall, the data show that the neuroprotective apoD is increased in AD brain tissue but not in FTLD, suggesting significantly different reactions to oxidative stress in these two forms of dementia—the apoD response in AD appears to be somewhat neuroprotective, while its unchanging level in FTLD may exacerbate the neurodegeneration.

## Electronic supplementary material

ESM 1 (DOCX 1934 kb)

## Abbreviations

apoD: Apolipoprotein D
FTLD: Frontotemporal lobar degeneration
AD: Alzheimer’s disease
PD: Parkinson’s disease
PSP: Progressive supranuclear palsy
CBD: Corticobasal degeneration
GGT: Globular glial taupathies
TBS: Tris-buffered saline
FBS: Fetal bovine serum
