# Supplementary material for: Apolipoprotein D Upregulation in Alzheimer’s Disease but Not Frontotemporal Dementia
Source: J Mol Neurosci. 2018 Nov 22;67(1):125–32. doi: 10.1007/s12031-018-1217-9 (PMC6344390; doi:10.1007/s12031-018-1217-9)
Supplement: Supplementary file 1 [file 12031_2018_1217_MOESM1_ESM.docx]

Fig 1

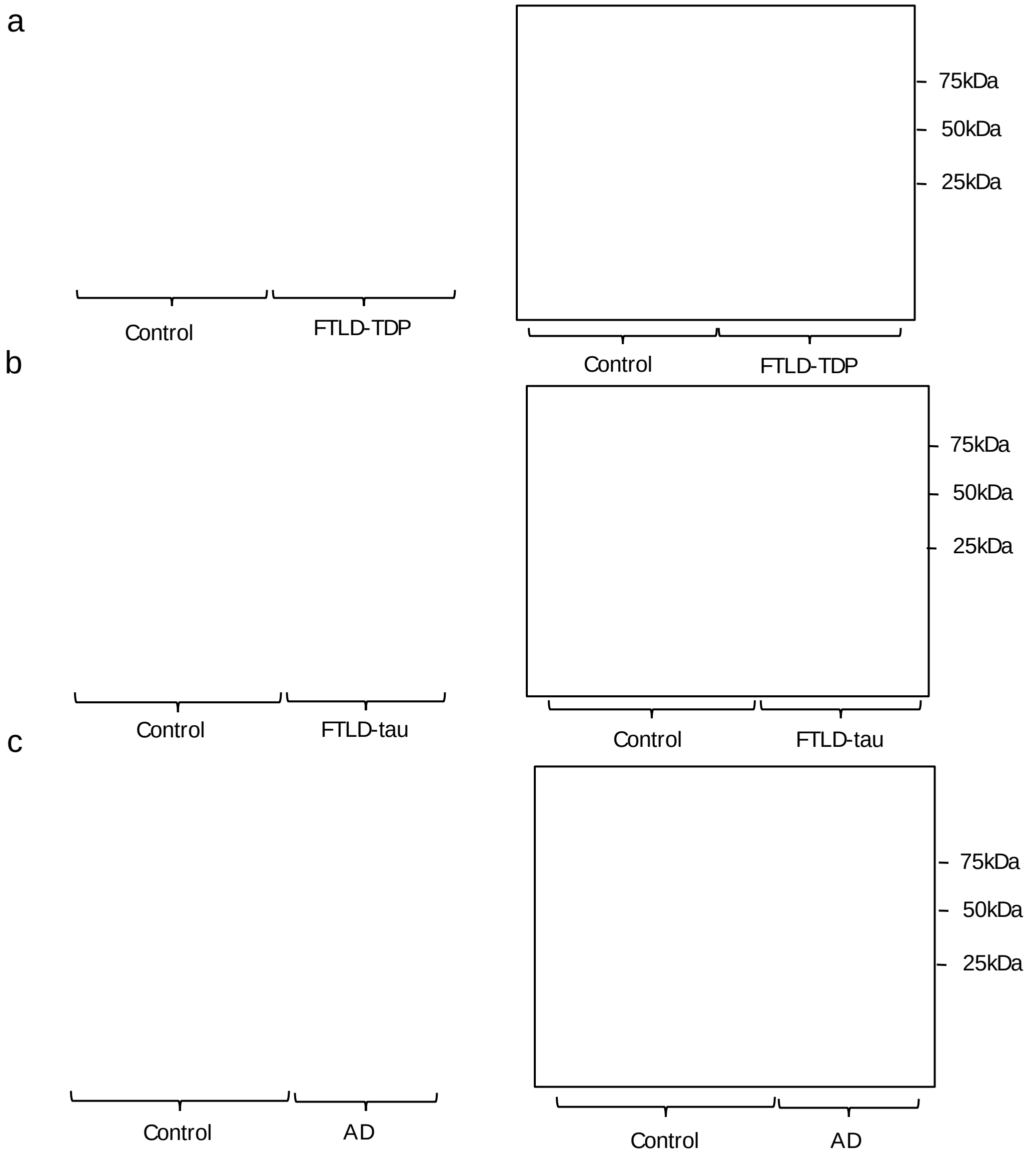

PVDF membrane after transfer from stain free gel

PVDF membrane probed for apoD

Fig.1 ApoD expression in SDS soluble brain fractions.

Fig 2

a

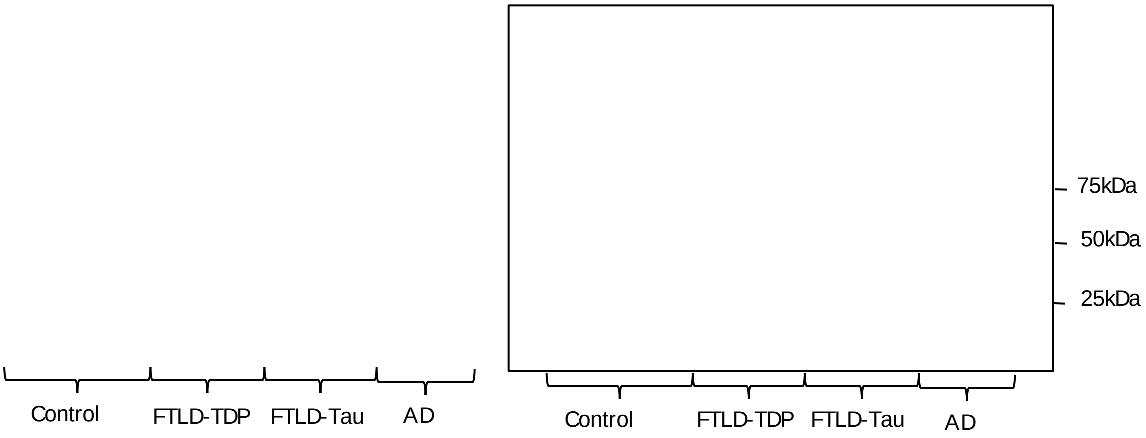

PVDF membrane after transfer from stain free gel

PVDF membrane probed for apoD

Fig.2 ApoD expression in SDS insoluble (gHCL soluble) brain fractions.

Fig 3

Fig.3 Cell viability assay for U87 and U87apoD cells at different conc. of hydrogen peroxide after 24 hour treatment. The Data represents Mean SEM (n=2 to 4 in triplicates).
